# An Improved Chest Wall Reconstruction Technique With Twisted Steel Wire–Reinforced Neoribs

**DOI:** 10.1016/j.atssr.2022.09.011

**Published:** 2022-09-24

**Authors:** Rabin Gerrah

**Affiliations:** 1 Department of Cardiothoracic Surgery, Stanford University, Stanford, California

## Abstract

**Purpose:**

Large chest wall defects often require reconstruction to improve the functionality of the chest wall and to achieve aesthetic results. An optimized technique for chest wall reconstruction with superior mechanical properties is presented here.

**Description:**

In this technique, the chest wall is reconstructed by neoribs. Synthetic neoribs are constructed by integration of twisted steel wires with bone cement, customized for the patient and without the exposure of cement to the medullary cavity of the ribs.

**Evaluation:**

The proposed technique is easy to reproduce, is low cost, and achieves excellent aesthetic results. Compared with other techniques, this technique achieves superior tensile and compressive strengths.

**Conclusions:**

Reconstruction of the chest wall with neoribs reinforced with twisted steel wire seems to be the optimal technique for chest wall reconstruction, with improved mechanical properties.

## Technology

Large chest wall resections create significant defects often requiring reconstruction. The chest wall, a dynamic structure composed of rigid and soft tissues, protects the vital organs and assists in breathing. Reconstruction after chest wall resection is important not only to achieve defect closure but also to preserve the mechanics of breathing. Multiple techniques using different materials have been described for chest wall reconstruction.^1^ The cornerstone of chest wall reconstruction has been covering the gap with various mesh or patch materials and combinations to achieve soft or rigid coverage of the gaps.^2^ These techniques achieve the sole purpose of defect coverage; however, they provide a nonanatomic repair by adding a static and often rigid patch to a dynamic structure. To address this issue, several techniques have been described to mimic the normal anatomy of the rib cage. Rigid bars and plates have been used as a rigid scaffold to replace the excised ribs,^1^ and synthetic neoribs that mimic the ribs have been introduced.^3^^,^^4^ The foundation for the neoribs has been polymethyl methacrylate (PMMA), commonly known as bone cement. PMMA obtains significant compressive strength, but it bears the disadvantage of low tensile strength and no adhesive properties. Although PMMA has been used in multiple applications for a long time, complications from direct exposure have been reported.^5^

An optimized technique of neorib construction with superior mechanical properties and elimination of PMMA exposure to the medullary cavity is described in this paper.

## Technique

After resection of the ribs, the defect is covered with an appropriately sized single and flexible polypropylene mesh, anchored to the perimeter of the defect on soft tissue and occasionally on the periosteum of the ribs to gain maximal strength, preventing lung herniation. This stage concludes the soft tissue coverage, and the rigid reconstruction commences.

**Figure 1:**
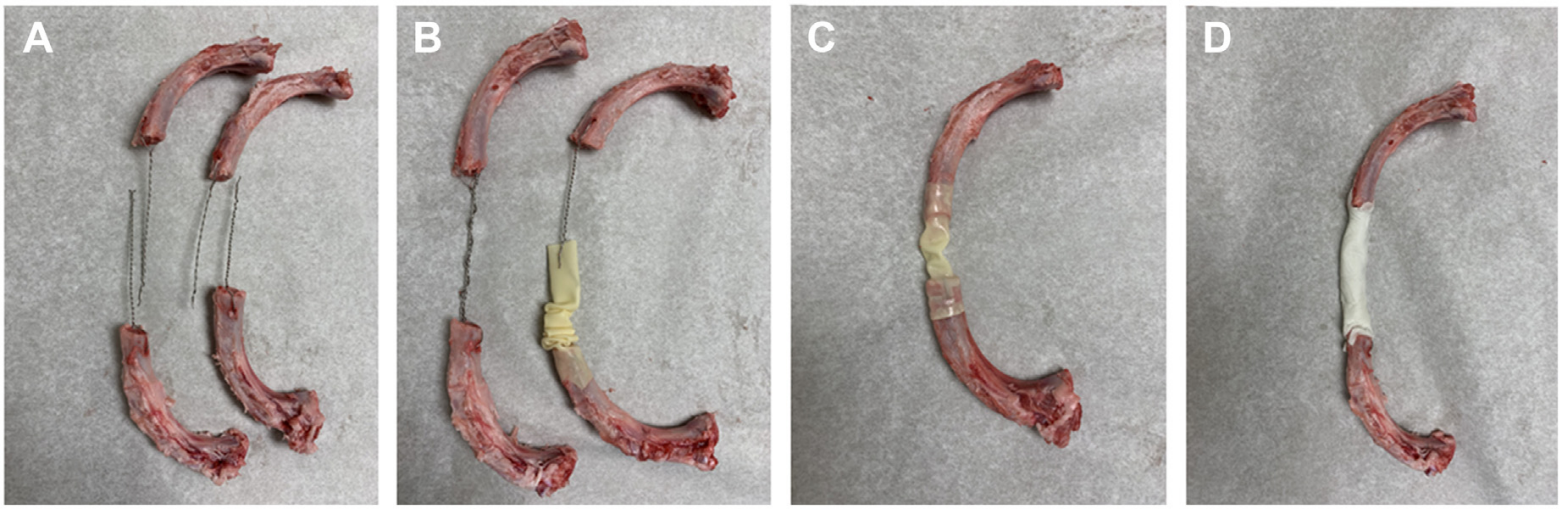
Demonstration of the technique on fresh porcine ribs. (A) Placement of wire through a drilled hole in the rib and twisted steel wire on each corresponding rib. (B) Twisted paired wires on each side (shown with and without the latex tube that functions as the molding structure). (C) Placement of the latex tube on each end after completion of the twisting and before injection of the bone cement inside the latex tube. (D) Final result of the reconstructed neorib after removal of the latex tubing.

Initially, a 1-mm-diameter hole is drilled across all resected rib ends, 1 to 1.5 cm from the tip of the rib. A USP No. 5 stainless steel wire (sternal wire) is passed through each hole and twisted on itself multiple times to achieve a twist density of 8 to 12 turns per inch. The twisted wires are cut long (approximately ¾ length of the defect), and a 0.5-inch-diameter soft latex tube, such as Penrose, cut 4 cm longer than the projected length of the defect, is passed over the twisted wires on 1 side only. This tube will be the molding structure of the neorib. The soft tube is pushed to 1 side to obtain maximal exposure of the twisted wires. The twisted wires on each corresponding rib on each side are partially twisted on each other this time, and the twist is shaped to the imaginary curvature of the resected rib. It is important that during wire twisting, the 2 ends are held by a clamp to stabilize the wires, preventing pull-through or damage on the rib ends. Care must be taken not to twist the wires too much as this will pull the ribs closer and will modify the anatomic shape. The latex tube is pulled over the entire length of the twisted wires, and the ends are ligated over the rib ends. A small hole is made in the middle of the tube, and cement is injected inside the tube, which acts as the neorib mold. Gentle compressions on the tube will help in uniform distribution and shaping of the neoribs. The cement is allowed to dry, and the latex tube and the ligatures are then cut and removed. The neorib construction is completed. The steps of this reconstruction are demonstrated on porcine ribs in Figure 1.

## Clinical Experience

**Figure 2:**
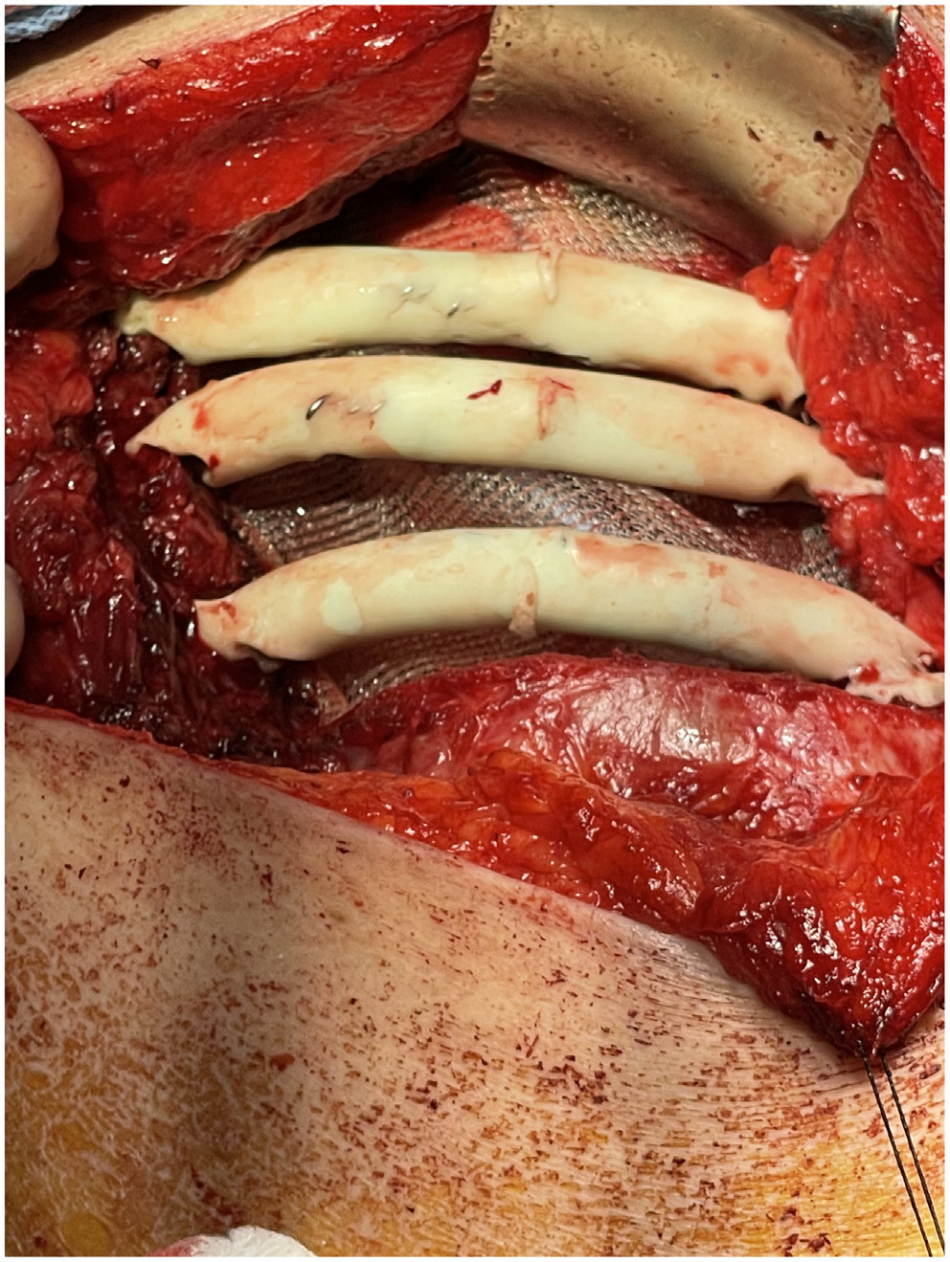
The end result of neorib reconstruction after bone cement hardening and removal of the latex tubing.

This technique was used to reconstruct the chest wall of a 72-year-old woman who presented with a single metastasis on the right seventh rib 1 year after right lower lobectomy for lung cancer. Wide resection of ribs 6, 7, and 8 was carried out, and it created a defect of 8 × 8 cm on the chest wall. Chest wall reconstruction by this optimized technique with 3 neoribs achieved excellent results (Figure 2). The patient had an unremarkable postoperative course and was discharged home on postoperative day 5. Postoperative 90-day follow-up showed an excellent functional and aesthetic outcome.

## Comment

In this paper, an improved chest reconstruction technique is described. Chest reconstruction with neoribs has been reported elsewhere. However, this technique has shown multiple advantages, mostly in mechanical properties; it improve the tensile strength of the neoribs, and it has the potential benefit of eliminating PMMA contact with intrathoracic organs or intramedullary content of the rib.

In general, reconstruction of the chest wall with neoribs has the advantage of maintaining dynamic properties of the rib cage and also achieving results visually mimicking the native anatomy. A neorib replacing a segment of the native rib is subject to the forces applied to the chest wall, mainly radial forces generated by chest wall expansion, projecting outward, and compressive forces from body weight applied on the chest in the supine position.

**Figure 3:**
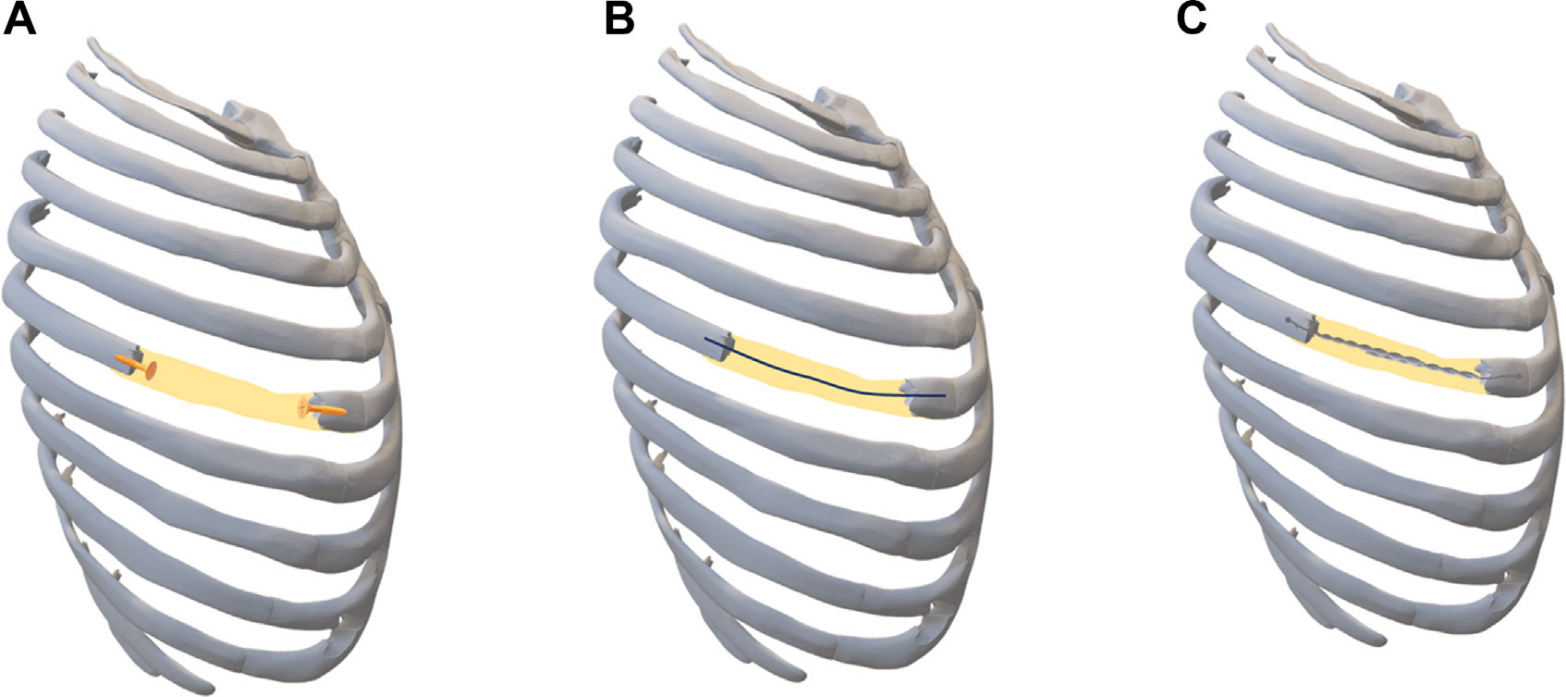
Schematic diagrams of different neorib reconstruction techniques. (A) Neorib is built on top of a structure, such as a pin or a screw, inserted in the rib end; bone cement is casted inside the flexible (yellow) tubing, as described.^3^ (B) Neorib reconstruction using a Steinmann pin on each end of the rib.^4^ (C) Neorib reconstruction using twisted steel wire anchored on each rib end, as described in this paper.

Lack of adhesive properties and the low tensile strength of PMMA require additional support structures to stabilize the ribs and the chest wall. In the initial work by Dahan and colleagues,^3^ the neorib is anchored by pins, whereas most of the neorib structure is made of PMMA only. Addition of a Steinmann pin in the PMMA of the neorib as described by Suzuki and coworkers^4^ improves compressive strength by adding a metal base in the cement, with no effect on the tensile strength of the neorib. It is assumed that integration of twisted steel wires in the cement reinforces it (similar to rebar rods in concrete), gaining significant compressive strength, and anchoring the wires to the ribs gains significant tensile strength. Improvement of the tensile strength of bone cement with twisted stainless steel wire has been reported.^6^ To compare the tensile and compressive strengths of the described technique with the other techniques, 2 models of each method (Figure 3) were built using fresh porcine ribs and subjected to compressive and tensile forces until the end point of fracture (Table). The reconstruction method with the twisted wires showed superior tensile and compressive strengths.

**Table:** Comparison of Fracture Load of the Porcine Neorib Samples Subjected to Compressive and Tensile Forces in 3 Reconstruction Techniques and Normal Ribs

	Neoribs Anchored to the Native Rib With Screws	Neoribs Anchored to the Native Rib With Steinmann Pins	Twisted Steel Wire–Reinforced Neoribs	Normal Ribs
Compressive force, kg	7	11	18	31
Tensile force, kg	52	36	>100	>100

This reconstruction technique is easily reproducible, is low in cost, and does not need any new hardware that might not be available in the thoracic surgery operating room. Moreover, there is no need for injection of the cement in the medullary space of the rib, therefore eliminating the potential complications of PMMA contact.

## Freedom of Investigation

The tested technology in this manuscript was not purchased, borrowed, or donated to this study. The author had full control of the design of the study, methods used, outcome parameters, analysis of data, and production of the written report.

## Disclaimer

The Society of Thoracic Surgeons, The Southern Thoracic Surgical Association, and *The Annals of Thoracic Surgery* neither endorse nor discourage the use of the new technology described in this article.
